# CROTON: an automated and variant-aware deep learning framework for predicting CRISPR/Cas9 editing outcomes

**DOI:** 10.1093/bioinformatics/btab268

**Published:** 2021-07-12

**Authors:** Victoria R Li, Zijun Zhang, Olga G Troyanskaya

**Affiliations:** Hunter College High School, New York, NY 10128, USA; Center for Computational Biology, Flatiron Institute, Simons Foundation, New York, NY 10010, USA; Center for Computational Biology, Flatiron Institute, Simons Foundation, New York, NY 10010, USA; Lewis-Sigler Institute for Integrative Genomics, Princeton University, Princeton, NJ 08540, USA; Department of Computer Science, Princeton University, Princeton, NJ 08544, USA

## Abstract

**Motivation:**

CRISPR/Cas9 is a revolutionary gene-editing technology that has been widely utilized in biology, biotechnology and medicine. CRISPR/Cas9 editing outcomes depend on local DNA sequences at the target site and are thus predictable. However, existing prediction methods are dependent on both feature and model engineering, which restricts their performance to existing knowledge about CRISPR/Cas9 editing.

**Results:**

Herein, deep multi-task convolutional neural networks (CNNs) and neural architecture search (NAS) were used to automate both feature and model engineering and create an end-to-end deep-learning framework, CROTON (CRISPR Outcomes Through cONvolutional neural networks). The CROTON model architecture was tuned automatically with NAS on a synthetic large-scale construct-based dataset and then tested on an independent primary T cell genomic editing dataset. CROTON outperformed existing expert-designed models and non-NAS CNNs in predicting 1 base pair insertion and deletion probability as well as deletion and frameshift frequency. Interpretation of CROTON revealed local sequence determinants for diverse editing outcomes. Finally, CROTON was utilized to assess how single nucleotide variants (SNVs) affect the genome editing outcomes of four clinically relevant target genes: the viral receptors *ACE2* and *CCR5* and the immune checkpoint inhibitors *CTLA4* and *PDCD1*. Large SNV-induced differences in CROTON predictions in these target genes suggest that SNVs should be taken into consideration when designing widely applicable gRNAs.

**Availability and implementation:**

https://github.com/vli31/CROTON.

**Supplementary information:**

Supplementary data are available at *Bioinformatics* online.

## 1 Introduction

Clustered regularly interspaced short palindromic repeats (CRISPR)/CRISPR-associated protein 9 (Cas9) is a revolutionary gene-editing technology that has broad applications in basic biology, biotechnology and medicine (Hsu *et al*., 2014). CRISPR/Cas9-mediated genome editing follows two major steps: (1) the induction of a double-stranded break (DSB) in a target DNA sequence and (2) the activation of cellular DNA-repair pathways. CRISPR/Cas9 is a ribonucleoprotein that consists of a guide RNA (gRNA) that defines a target DNA sequence and the dual DNA endonuclease Cas9 which induces a DSB around 3 base pairs (bps) upstream of an ‘NGG’ protospacer adjacent motif (PAM). Following DNA cleavage, a DSB can be repaired by three DNA repair pathways: template-free non-homologous end-joining (NHEJ) and microhomology-mediated end joining (MMEJ), as well as template-directed homology-directed repair (HDR). HDR can be used to introduce precise DNA modifications, but it is inefficient, especially in non-mitotic cells, and often generates unwanted byproducts. In contrast, NHEJ and MMEJ were believed to trigger random repair outcomes. However, recent research has shown that NHEJ and MMEJ repair outcomes are dependent on features on target DNA sequences (Molla and Yang, 2020).

Since a range of DNA sequence factors, such as GC content and microhomology length and position, may contribute to repair outcomes, accurate prediction of template-free CRISPR/Cas9 editing outcomes is a challenging bioinformatics question. Three machine learning (ML) models, inDelphi, FORECasT and SPROUT, which utilize neural networks and k-nearest neighbors, multinomial logistic regression, as well as gradient-boosting decision trees, respectively, have been designed to tackle this question. However, these ML methods require both feature and model engineering and are thus limited by existing knowledge about CRISPR/Cas9-induced DSB repair (Allen *et al*., 2019; Leenay *et al*., 2019; Shen *et al*., 2018).

A potential alternative ML framework is deep convolutional neural networks (CNNs), which have attracted attention in computational biology because they excel at pattern recognition. Indeed, many state-of-the-art ML models capable of predicting specific molecular phenotypes from raw DNA sequences utilize deep CNNs (Eraslan *et al*., 2019; Zhang *et al*., 2021). Since deep CNNs process raw sequences, manual feature engineering is not required for CNN-generation, which can expedite model creation. Furthermore, CNNs can detect and process important, but not well-understood, parts of an input, rendering it potentially more effective and versatile relative to other ML methods. Effective model architectures are essential for CNN performance, but CNN architecture design requires a substantial amount of ML knowledge and time. Recently, neural architecture search (NAS), a state-of-the-art method for finding good neural network architectures has been developed to automate model-engineering. NAS is a form of automated machine learning (AutoML) that has been shown to generate CNNs with comparable efficacy to manually engineered models (Zhang *et al.*, 2021; Zoph and Le, 2017).

**Fig. 1.**
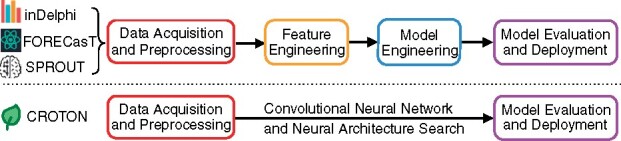
The CROTON ML pipeline is highly automated. Unlike the three existing models for CRISPR/Cas9 editing outcome prediction, CNN and NAS-based CROTON is based on automated feature and model design, which creates an end-to-end ML pipeline from data acquisition to model deployment

Herein, CROTON (CRISPR Outcomes Through cONvolutional neural networks), a novel deep learning framework based on deep CNNs and NAS, has been created to predict CRISPR/Cas9 editing outcomes. By leveraging CNNs and NAS, CROTON fully automates the tasks of predicting 1 bp insertion and deletion probability as well as deletion and frameshift frequency from raw sequences alone and without any prior knowledge (Fig. 1). We demonstrate that CROTON, which was trained on a synthetic construct-based dataset, outperforms existing approaches on a held-out, independent endogenous T-cell dataset. CROTON was then utilized to evaluate the effect of single nucleotide variants (SNVs) on the CRISPR/Cas9-mediated genome editing outcomes of four clinically relevant target genes: *ACE2*, *CCR5*, *CTLA4* and *PDCD1*. The differences in predicted SNV-induced editing outcomes suggest that SNVs should be considered when designing widely applicable gRNAs.

## 2 Materials and methods

### 2.1 Acquisition and pre-processing of CRISPR/Cas9 editing outcome datasets

The datasets used to train CROTON were acquired from two previous works that produced the models FORECasT and SPROUT (Allen *et al.*, 2019; Leenay *et al.*, 2019). To reconcile the two datasets, we compiled 60 bp genomic sequences as the model inputs. Specifically, for each gRNA in the FORECasT dataset, we aligned the PAM sites at 33 nt so the cut site was at the center (30 nt) of all input sequences. The pseudo-letter ‘N’ was padded to the FORECasT sequences if they were shorter than 60 bp after PAM realignment. To obtain DNA sequences for SPROUT, retrieved genomic coordinates were mapped to the human genome build 38 (hg38). Subsequently, sequences from FORECasT and SPROUT were one hot encoded to 4×n matrices for each DNA sequence, where *n *=* *60 was the sequence length, the nucleotide ‘A’ was represented by the array [1, 0, 0, 0], ‘C’ was represented by [0, 1, 0, 0], ‘G’ was represented by [0, 0, 1, 0], ‘T’ was represented by [0, 0, 0, 1] and ‘N’ was represented by [0.25, 0.25, 0.25, 0.25].

To compile the editing outcomes, CIGAR (Compact Idiosyncratic Gapped Alignment Report) strings were processed for the FORECasT and SPROUT datasets. For each gRNA, we computed the following editing outcome statistics: (1) 1 bp insertion frequency, (2) 1 bp deletion frequency, (3) deletion frequency, (4) 1 bp frameshift frequency, (5) 2 bp frameshift frequency and (6) total frameshift frequency. (Given *I* is the total number of insertions, *D* is the total number of deletions and *I *+* D* is the total number of insertions or deletions (indels), the first three metrics were defined as follows: (i) $\frac{I_{1bp}}{I+D},$ (ii) $\frac{D_{1bp}}{I+D}$ and (iii) $\frac{D}{I+D}$. The next three frameshift frequency statistics were defined as the proportion of indel outcomes that induced a frameshift of 1 bp, 2 bp or the union of both).

We leveraged the large-scale FORECasT data to train the model and held out the SPROUT dataset as an independent dataset for performance evaluation. Within the FORECasT data, samples were randomly split into training, validation and testing datasets in an 8:1:1 ratio. The FORECasT training dataset had 28 105 datapoints, and both the FORECasT test and validation datasets had 3512 datapoints. In addition, the SPROUT dataset, which we utilized for cross-cellular testing, had 1603 datapoints. The validation dataset was used to monitor model training convergence and early-stopping, while the testing datasets were held-out as independent, unseen datasets to evaluate the trained model performance.

### 2.2 Automated deep learning interface for CRISPR/Cas9 editing outcome prediction

CROTON is a deep CNN that predicts CRISPR/Cas9 editing outcomes from raw one hot encoded DNA sequences. Given a one-hot encoded input sequence of shape $x_{i}\in R^{4\times 60}$, the task for CROTON was to learn a function $f_{\omega ;a}(\cdot )$ with trainable parameters *ω* under a fixed architecture *a* that mapped a sequence *x_i_* to a vector of six indel and frameshift-related probabilities $y_{i}=\{y_{ij}\in [0,1]|j=1,2,\ldots ,6\}$, such that $y_{i}=f_{\omega ;a}(x_{i})$. To search for expressive architectures *a* and learn $f_{\omega ;a}$, AMBER (v0.1.0), a framework for CNN architecture design for genomic sequence processing, was utilized to automatically design the CROTON model architecture. In AMBER, CROTON’s input and output stems were fixed to fit the input sequences and output labels, while its middle eight convolution layers were searched (Zhang *et al.*, 2021).

We first describe the fixed input and output stems for CROTON. The input layer contained a 4 × 60 matrix *x_i_*, followed by a linear stem convolution layer with kernel size 8 that expanded the 4-channel DNA sequence into 32 channels. The top of the model employed global average pooling that flattened convolution layers to a fully connected layer with 32 hidden units, and the final outputs of the model were multi-tasking predictions for each of the six editing outcome statistics. We used binary cross-entropy as the loss function to update *ω* for predictions of the six indel and frameshift-related probabilities on a set of *N* training datapoints:

$$L(\omega ;x,y)=\frac{1}{N}\sum_{i=1}^{N}y_{i}^{T}\cdot \;\text{log}(f_{\omega ;a}(x_{i}))+{\left(1-y_{i}\right)}^{T}\cdot \;\text{log}(1-f_{\omega ;a}(x_{i})).$$

Next, we describe the model search space for the variable layers of CROTON. Specifically, the middle eight convolution layers were variable, and their computational operations and residual connections were searched by AMBER (Zhang *et al.*, 2021) to build an optimal CNN architecture. For each layer, AMBER searched for six candidate computational operations: four convolution layers with Rectified Linear Unit (ReLU) activation, kernel size ∈{4,8} and dilation rate ∈{1,4}, as well as the maximum and average pooling layers with pooling size = 4 and stride size = 1. Convolution operations across all layers had 32 filters, consistent with the input stem linear convolution. A special operation, identity mapping, was also added at each layer to potentially reduce model complexity. For any layer *t*, the computation operation was sparsely encoded by $a_{t}^{o}\in [1,7]$. Residual connections for the *t*th layer were encoded as binary tokens $a_{t}^{r}\in \{0,1\}$ from each of the preceding layers 1,2,..,t−1. For brevity, we let $a=\{a_{t}^{o},a_{t}^{r}|t=1,2,\ldots ,T\}$ be CROTON’s model architecture tokens for both computation operations and residual connections in the *T *=* *8 model space, such that a set of architecture tokens *a* fully specifies a model architecture for CROTON. In total, this eight-layer model space hosted $1.54\times 10^{15}$ viable model architectures.

Therefore, the architecture search problem was formulated as a sparse classification for the selection of computation operations, and binary classifications for residual connections, respectively. AMBER leverages a recurrent neural network (RNN) with parameters *θ* as a controller model to generate CROTON’s model architectures *a* with log-likelihood π(a;θ). A detailed explanation of the AMBER workflow can be found in our published work (Zhang *et al.*, 2021).

Formally, let $a_{t}^{o}$ denote the computation operation, and $a_{t}^{r}$ denote residual connections for the *t*th layer; let *h_t_* denote the hidden states of the controller model at the *t*th layer. At each layer *t*, $a_{t}^{o}$ and $a_{t}^{r}$ were sampled probabilistically from multinomial and binomial distributions, respectively; subsequently, the sampled tokens were fed as inputs to the next layer *t *+* *1. In particular, the controller model predicted the $a_{t}^{o}$ by first updating the hidden state through a long short-term memory (LSTM) cell $h_{t}=f_{\theta_{o}}(a_{t-1}^{o};h_{t-1})$, then sampling from the multinomial distribution of softmax function σ(·) transformed *h_t_* by weight *W_o_*:

$$P(a_{t}^{o})=\sigma (W_{o}\cdot f_{\theta_{o}}(a_{t-1}^{o};h_{t-1}))=\sigma (W_{o}\cdot h_{t}).$$

The residual connection for the *t*th layer from the *r*th layer, 1≤r<t, was sampled from the binomial distribution whose probability was determined by an attention mechanism between the query layer’s hidden state *h_t_* and the previous layer’s hidden state *h_r_*, with trainable weights *v*, $W_{r1}$ and $W_{r2}$:

$$P(a_{t}^{r})=\sigma (v^{T}\cdot \tanh(W_{r1}\cdot h_{t}+W_{r2}\cdot h_{r})).$$

Thus, the total trainable parameters for the controller model were $\theta =\{\theta_{o},W_{o},W_{r1},W_{r2},v\}$, and the log-likelihood for selecting a set of architecture tokens *a* under the parameters *θ* was π(a;θ). We employed reinforcement learning to optimize *θ*. Following the previously established REINFORCE rule (Williams, 1992), the policy gradient for *θ* was obtained to maximize the average multi-tasking Spearman’s correlation coefficient R on the validation dataset over a batch of *m* sampled architectures, with an exponential moving average of rewards *b* to stabilize the reward signals:

$$\frac{1}{m}\sum_{k=1}^{m}\nabla_{\theta }\pi (a_{k};\theta )(R_{k}-b).$$

Finally, the optimal AMBER-searched CNN architecture, which was defined as the best reward architecture in the last controller step, was scaled in width by a scaling factor. Dropouts were then added after each searched layer before the architecture was re-trained from scratch. We performed a simple grid search for these two additional hyperparameters and reported the best performing model with width scaling factor = 6 and dropout rate = 0.4. Training convergence was defined as validation loss not decreasing for at least 50 epochs.

### 2.3 Performance comparisons

We sampled CNN architectures from the same model space without training the AMBER controller model to benchmark the quality of the automatically designed model architecture. In particular, computational operations were sampled uniformly from the model space; residual connections were sampled at the same density as CROTON. A total of *n *=* *50 models with sampled architectures were trained with identical width-scale factor and optimization configurations to robustly evaluate an uninformed, null distribution of performance in the model space. Subsequently, the testing performance for every CROTON prediction task was compared to that of the sampled model cohort.

We also evaluated CROTON’s predictions by classifying each individual task’s predicted probability as high (larger than the observed median value) versus low (lower than the observed median value), and calculated area under the curve receiver operating characteristics (AUC-ROC) for this binary classification problem.

Furthermore, we applied the trained CROTON model on the held-out SPROUT T-cell dataset as an independent, cross-cellular benchmark. Existing methods were benchmarked against CROTON, including inDelphi (Shen *et al.*, 2018), FORECasT (Allen *et al.*, 2019) and SPROUT (Leenay *et al.*, 2019). For inDelphi and FORECasT, we used the publicly available trained models (https://github.com/maxwshen/inDelphi-model and https://github.com/felicityallen/SelfTarget) to generate predictions for all sequences in the SPROUT dataset. The Pearson’s correlation between predicted and observed values was then utilized to compare the performance of inDelphi and FORECasT to that of CROTON. Since inDelphi had different models for different cell lines, we reported values from the best performing inDelphi cell-line/model. For the SPROUT model trained on the SPROUT dataset, we compared CROTON’s performance to the published metrics (Leenay *et al.*, 2019) under the criteria defined by SPROUT (i.e. Kendall’s tau for 1 bp insertion and deletion probabilities, and Pearson’s correlation for deletion frequency).

### 2.4 *In silico* saturated mutagenesis analysis for model interpretation

To interpret how the CNNs made their predictions, *in silico* saturated mutagenesis was performed using the Selene framework (Chen *et al*., 2019). *In silico* saturated mutagenesis is a perturbation-based base importance analysis method in which CNNs evaluate DNA sequences with single nucleotide polymorphisms (SNPs). In an SNP, a nucleotide at a specific position along a DNA sequence is changed to another, for instance, ‘ACC’ is a perturbed sequence of ‘GCC’. In *in silico* saturated mutagenesis, the model runs on every possible one hot encoded sequence that can be perturbed from the original sequence. The final interpretation output is a matrix with the same shape as the input (4 × 60) in which every matrix entry represents a base importance score calculated as the difference between the predictions of the reference sequence and the altered sequence. In summary, *in silico* saturated mutagenesis evaluates how important every base pair position is to a CNN by computing the deviation of its predictions for sequences with SNPs at that position from the original unperturbed sequence. Herein, sequences with model predictions within 0.05 of true values were utilized for *in silico* saturated mutagenesis analysis.

### 2.5 Variant effect analysis for frameshift gRNA design

The human genome-wide variants dbSNP build 151 VCF file was downloaded from NCBI (ftp.ncbi.nih.gov/snp/organisms/human_9606_b151_GRCh38p7/VCF/). For all annotated coding exons in Gencode V35, we scanned potential PAM sites (‘NGG’) in the hg38 genome before aligning them to the CROTON 60 bp window. Then, bedtools (v2.29) was used to intersect the PAM sequences to the variants. For each PAM site with variants in the four representative genes (*ACE2*, *CCR5*, *CTLA4* and *PDCD1*), CROTON predicted editing outcome probabilities for sequences with reference and alternative alleles. The differences between reference and alternative alleles were subsequently calculated for each of the individual tasks. In addition, to find the least variant gene-editing targets, the absolute differences between the reference and alternative CROTON predictions were computed across all statistics for all SNVs at a particular potential target location. Then these targets were ranked by the mean of their absolute differences to elucidate the gene targets with the least impactful SNVs.

## 3 Results

### 3.1 Automated model architecture design for CROTON

CROTON was built on data from FORECasT because it produced the largest CRISPR/Cas9 editing outcome dataset relative to those of inDelphi and SPROUT (Allen *et al.*, 2019; Leenay *et al.*, 2019; Shen *et al.*, 2018). FORECasT data was split into training, validation and testing datasets and all metrics presented are from model performance on the testing dataset, which CROTON was unexposed to during training. CROTON was designed to predict 1 bp insertion and 1 bp deletion probability, as well as deletion, 1 bp frameshift, 2 bp frameshift and overall frameshift frequency. Since these features were interrelated, we chose to utilize a multi-task learning framework.

**Fig. 2.**
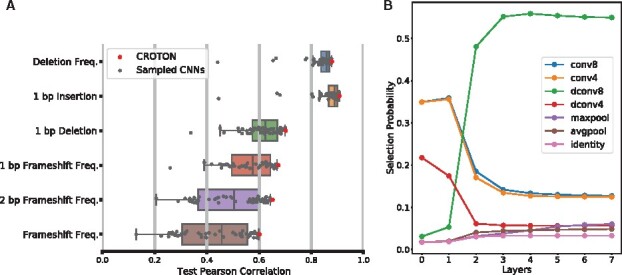
NAS designs effective multi-task deep CNN architectures. (**A**) CROTON outperforms models with sampled architectures from the model search space. CROTON achieves *R_P_* of > 60 for all prediction tasks and *R_P_* of 87.96 and 90.79 for deletion frequency and 1 bp insertion probability prediction, respectively. (**B**) The layer selection probabilities for the best CROTON architecture

Multi-task learning can outperform single-task learning by leveraging features derived for multiple prediction tasks (Zhang and Yang, 2018). In addition, manually tuning a CNN would be time-consuming and limited in scope. Thus, we utilized NAS to automatically create a multi-task deep CNN framework for CRISPR/Cas9 editing outcome prediction. The NAS model search space contained dilated and non-dilated one-dimensional convolutional layers with kernel sizes four and eight (dconv4, dconv8, conv4 and conv8) as well as the maximum pooling (maxpool), average pooling (avgpool) and identity layers (Methods). To assess the efficacy of automated model engineering, the final NAS architecture was compared to 50 randomly sampled model architectures from the search space. The final NAS-designed CROTON model outperformed all randomly selected model architectures, indicating that NAS is an effective strategy for deep-CNN design. The final CROTON architecture achieved Pearson’s Correlations (*R_P_*) greater than 60 for all prediction tasks and *R_P_* greater than 70 for deletion frequency, 1 bp insertion probability and 1 bp deletion probability prediction (Fig. 2A).

We also analyzed the average layer selection probabilities for the NAS run. Interestingly, across all model layers, convolutional layers were consistently favored over pooling layers, indicating that precise feature locations were conserved in our model. In addition, a dilated convolutional layer of size 8 was favored for all layers after Layer 1. Dilated layers allow the receptive field to be enlarged without losing resolution or coverage, further suggesting that spatial relationships between features were important for CRISPR/Cas9 outcome prediction (Fig. 2B;  Yu and Koltun, 2016).

### 3.2 Croton accurately predicts CRISPR/Cas9 editing outcomes across cell lines and outperforms other predictors

**Fig. 3.**
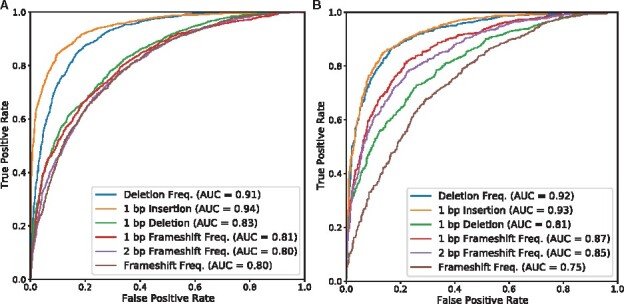
CROTON makes accurate CRISPR/Cas9 editing outcome predictions across cell lines. (**A**) AUC-ROC curves for CROTON’s predictions on the testing FORECasT dataset. (**B**) AUC-ROC curves for CROTON’s performance on the held-out, primary T cell-derived SPROUT dataset. Across cell lines, CROTON achieved AUCs > 0.75 for all prediction tasks and AUCs > 0.90 for deletion frequency and 1 bp insertion prediction

The efficacy of CROTON was also assessed by computing whether it made accurate predictions above or below the median value in each task dataset. Using this evaluation strategy, the area under the curve (AUC) was calculated to measure CROTON’s performance. On the FORECasT data, CROTON achieved AUCs of greater than 80 for all prediction tasks and greater than 90 for the deletion frequency and 1 bp insertion tasks (Fig. 3A). Since FORECasT data was based on synthetic gRNA-CRISPR target constructs, it was important to test CROTON on an endogenously generated gene-editing dataset (Allen *et al.*, 2019). To this end, CROTON was tested with the held-out, independent SPROUT CRISPR/Cas9 editing outcome dataset. This dataset was derived from primary human T cells, which are widely utilized in therapeutic cell engineering (Leenay *et al.*, 2019; Wang *et al*., 2020). On the SPROUT dataset, CROTON’s performance was conserved with AUCs similar to those measured with the FORECasT dataset, indicating that large-scale synthetic construct based datasets are effective for endogenous CRISPR/Cas9 predictions (Fig. 3B).

CROTON’s predictive accuracy on the SPROUT dataset was then compared to that of existing ML-based CRISPR/Cas9 editing outcome predictors: SPROUT, FORECasT and inDelphi. For inDelphi, metrics for the best performing model on the HEK293 cell line were reported. Overall, CROTON substantially outperformed all models on all but one task. CROTON was only less effective than FORECasT at frameshift frequency prediction but outperformed FORECasT with wide margins on other prediction tasks such as deletion and 1 bp insertion frequency (Tables 1 and 2). Notably, CROTON performed on par or even outperformed SPROUT, which was trained on the SPROUT dataset.

**Table 1.** Performance comparison of CROTON, inDelphi and FORECasT by Pearson’s correlation (*R_P_*)

	CROTON	inDelphi	FORECasT
Deletion frequency	**81.12**	51.00	73.17
1 bp insertion	**82.42**	52.40	75.10
1 bp deletion	**57.51**	21.45	30.36
1 bp frameshift frequency	**73.84**	54.69	66.71
2 bp frameshift frequency	**64.30**	42.40	50.04
Frameshift frequency	55.56	51.54	**57.94**

**Table 2.** Performance comparison of CROTON and SPROUT

	CROTON	SPROUT
Deletion frequency (*R_P_*)	**81.12**	77
1 bp insertion (K Tau)	**65.22**	62
1 bp deletion (K Tau)	**43.81**	40

### 3.3 *In silico* mutagenesis revealed local sequence determinants for diverse editing outcomes

**Fig. 4.**
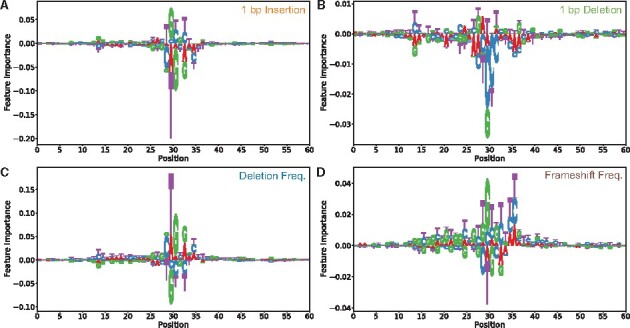
The importance assigned by CROTON to every nucleotide on the input sequence *In silico* saturated mutagenesis plots for 1 bp insertion probability (**A**), 1 bp deletion probability (**B**), deletion frequency (**C**) and frameshift frequency (**D**)

Since CROTON is an effective CRISPR/Cas9 editing outcome predictor and does not utilize any manual feature engineering, it was important to elucidate how CROTON made predictions from a raw input sequence. Thus, we conducted *in silico* saturated mutagenesis for CROTON on all prediction tasks for both the FORECasT and SPROUT datasets. These plots display the average importance values computed over multiple sequences in these test datasets (Methods). Across FORECasT and SPROUT data, saturated mutagenesis plots for the same prediction task were very similar. Representative *in silico* saturated mutagenesis plots based on FORECasT data are shown in which larger text is indicative of nucleotides with greater importance to CROTON prediction (Fig. 4).

Consistent with prior reports, the base pairs upstream of the PAM sequence were the most important for CROTON’s template-free CRISPR/Cas9 editing outcome predictions (Fig. 4; Leenay *et al.*, 2019; Shen *et al.*, 2018). In particular, our analyses support cross-cell line and cross-organism studies that have shown that the nucleotide 4 base pairs upstream of the PAM has the greatest effect on CRISPR/Cas9 DSB repair (Fig. 4A and C; Molla and Yang, 2020). Thus, our study confirms that the positions of nucleotides relative to the PAM site are important to CRISPR/Cas9 editing outcomes. Notably, 1 bp deletion and frameshift frequency had determinants across the entire input sequence (Fig. 4B and D), suggesting that they are more complex prediction tasks.

### 3.4 Croton reveals the effects of SNVs on CRISPR/Cas9-mediated genome editing

There are approximately 10–15 million common human SNVs, which can impact the efficacy of CRISPR/Cas9 editing (Chen *et al*., 2020; Eichler *et al*., 2007). Since CROTON accurately predicts 1 bp insertion probability with the best performance (Tables 1 and 2), we utilized 1 bp insertion probability to analyze the effect of SNVs on CRISPR/Cas9 editing outcomes. CROTON was applied across the coding regions of the gene bodies of 4 clinically relevant gene editing targets: *ACE2*, *CCR5*, *CTLA4* and *PDCD1*. *ACE2* and *CCR5* are receptors for the SARS-CoV-2 virus and the human immunodeficiency virus (HIV), respectively, and have been considered as therapeutic targets for viral infection (Michauld *et al*., 2020; Vangelista and Vento, 2018). *CTLA4* and *PDCD1* are immune checkpoint inhibitors that can be targeted for cancer immunotherapy (Shi *et al*., 2017; Stadtmaue *et al*., 2020; Wang *et al.*, 2020). Indeed, several ongoing clinical trials utilize CRISPR/Cas9 to delete *PDCD1*, including one that has been deemed safe and feasible for late-stage non-small cell lung cancer (NSCLC) patients (ClinicalTrials.gov NCT02793856; Lu *et al*., 2020; Wang *et al.*, 2020).

**Fig. 5.**
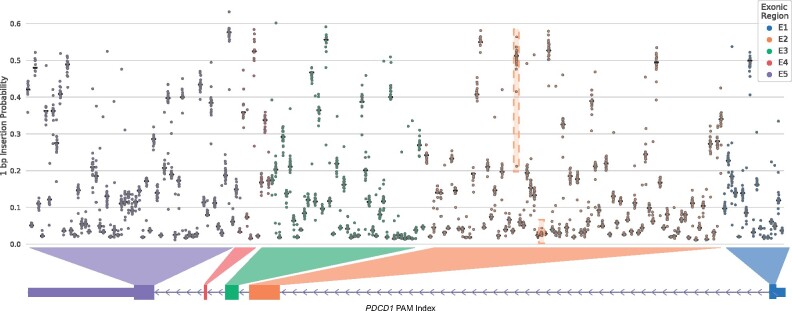
SNVs affect CRISPR/Cas9 editing outcomes. The distribution of CROTON’s 1 bp insertion probability predictions on all 211 PAM sites across the five *PDCD1* coding regions. The orange boxes indicate gRNA1 (left) and gRNA2 (right), which were utilized in an NSCLC clinical trial that used CRISPR/Cas9 to inactivate *PDCD1*. The black horizontal line indicates the 1 bp insertion probability prediction for the reference sequence, while circles (color-coded by exon) indicate the 1 bp insertion probability predictions for sequences with alternative alleles

Notably, CROTON’s analysis revealed that there were SNVs that altered the 1 bp insertion probability by ≥ 30% in all four clinically relevant genes (Table 3). We have also tabulated the top ten least variant gene target locations for each of these genes (Supplementary Tables S1–S4). Since 1 bp insertions result in frameshift mutations that will likely inactivate the target gene, these findings indicate that personalized genomic variants should be properly considered for these PAM sites for genome-editing applications in patients. We further analyzed *PDCD1* because it is involved in the greatest number of ongoing interventional CRISPR/Cas9 clinical trials (Wang *et al.*, 2020). 1 bp insertion probability in *PDCD1* varies considerably across the coding regions of the gene body. Notably, the two gRNAs which were used in the NSCLC trial, hereafter referred to as gRNA1 and gRNA2, had a high and low 1 bp insertion probability, respectively (Fig. 5; boxed in orange). These differences indicate that gRNA1 by itself is more likely to create a loss of function mediated by a 1 bp insertion than gRNA2. CROTON’s predictions indicate that SNVs may be important factors to consider in CRISPR/Cas9 genome editing, especially in clinical trials with patients that harbor these variants.

**Table 3.** SNVs with a High Impact on 1 bp Insertion Probability

Gene	Variant	Reference Pred.	Alternate Pred.	Absolute difference
*PDCD1*	rs1284638279	0.576	0.110	0.466
*ACE2*	rs1482922566	0.656	0.222	0.434
*ACE2*	rs370610075	0.056	0.489	0.432
*PDCD1*	rs535799968	0.029	0.429	0.399
*PDCD1*	rs141119263	0.202	0.601	0.398
*PDCD1*	rs769685838	0.130	0.524	0.394
*PDCD1*	rs371902970	0.132	0.515	0.382
*PDCD1*	rs370660750	0.116	0.497	0.381
*PDCD1*	rs1021665035	0.110	0.475	0.365
*PDCD1*	rs1185044781	0.399	0.036	0.363
*CCR5*	rs1032906612	0.060	0.422	0.362
*CCR5*	rs139737901	0.190	0.552	0.362
*CCR5*	rs767205045	0.546	0.186	0.360
*PDCD1*	rs368550965	0.184	0.537	0.353
*PDCD1*	rs749023157	0.039	0.388	0.350
*CCR5*	rs768195565	0.583	0.248	0.336
*CTLA4*	rs1461208141	0.420	0.098	0.322
*ACE2*	rs148036434	0.472	0.149	0.322
*PDCD1*	rs1422265917	0.015	0.336	0.321
*ACE2*	rs748076875	0.077	0.393	0.317
*CTLA4*	rs1444367175	0.221	0.537	0.316
*ACE2*	rs1395782023	0.083	0.398	0.314
*PDCD1*	rs146642159	0.033	0.346	0.313
*PDCD1*	rs1485118790	0.389	0.080	0.309
*PDCD1*	rs1329281649	0.398	0.095	0.303
*PDCD1*	rs774374376	0.019	0.321	0.302
*PDCD1*	rs1371267560	0.512	0.212	0.300

## 4 Discussion

CRISPR/Cas9 is a transformative gene-editing technology that has been widely applied in basic and translational biological research (Hsu *et al.*, 2014; Wang *et al.*, 2020). Recently, the creation of ML models capable of predicting the repair outcomes of CRISPR/Cas9 editing has highlighted the potential of predictable and precise template-free genome editing paradigms (Molla and Yang, 2020). However, existing ML prediction methods are all dependent on feature and model engineering, which may restrict their performance to current knowledge about CRISPR/Cas9 editing (Allen *et al.*, 2019; Leenay *et al.*, 2019; Shen *et al.*, 2018). Notably, deep CNNs and state-of-the-art NAS have been used to generate computational models based on genomic sequences (Eraslan *et al.*, 2019; Zhang *et al.*, 2021; Zoph and Le, 2017). In this study, we created CROTON, a novel framework that leverages both multi-tasking deep CNNs and NAS to predict CRISPR/Cas9 editing outcomes. CROTON predicts 1 bp insertion and 1 bp deletion probability, as well as deletion, 1 bp frameshift, 2 bp frameshift, and overall frameshift frequency directly from raw DNA target sequences. CROTON is highly automated relative to existing ML prediction methods and it outperforms them on a primary T cell-based genomic editing dataset. These results highlight the potential for CNNs and NAS for the precise prediction of genomic editing outcomes. A CROTON web interface has been made publicly available at the following link: https://github.com/vli31/CROTON.

The efficacy of NAS-designed models implies that NAS has significant potential in genomics and can design models that accurately predict molecular phenotypes from raw sequence alone. Furthermore, *in silico* saturated mutagenesis of CROTON showed that nucleotides upstream of the PAM were important to CNN prediction, which aligns with previous reports (Molla and Yang, 2020). Currently, although CROTON was built on the synthetic construct-based FORECasT dataset, when tested on the endogenous genomic SPROUT dataset, CROTON’s accuracy was largely conserved and it outperformed existing models. CROTON’s effectiveness between these two datasets indicates that utilizing synthetic constructs is an effective strategy to generate the large-scale data necessary for ML.

CROTON is highly effective in predicting 1 bp insertion probability, which can result in a frameshift mutation that inactivates a target gene. However, similar to other CRISPR/Cas9 editing outcomes predictors, CROTON is less effective in predicting overall frameshift frequency, which may limit its usage for loss-of-function gRNA design. CROTON’s accurate 1 bp insertion probability predictions were applied to 4 clinically relevant target genes to assess how SNVs affect genome editing outcomes. On all four genes, *ACE2*, *CCR5*, *CTLA4* and *PDCD1*, CROTON found variants that caused a significant difference in 1 bp insertion probability. To our knowledge, this is the first study that considers how naturally occuring variants affect CRISPR/Cas9 gene editing outcomes. We found that genomic loci with SNVs that have large effects on CRISPR/Cas9 editing outcomes should be avoided in widely applicable gRNA design. Further analysis of two gRNAs that were utilized in an NSCLC clinical trial revealed differential 1 bp insertion probability. Future studies may reveal whether this difference has a significant impact on genome editing outcomes in patients and whether there are better gRNA pairs for effective *PDCD1* genome editing. A CRISPR editing outcome predictor sensitive to genetic alterations at base-pair resolution like CROTON could be critical for designing effective gene therapies tailored to individual patients.

In addition, CROTON may be further developed to predict a more complete spectrum of DNA repair sequences. Notably, template-free CRISPR/Cas9-based correction of genetic diseases has been performed in Hermansky-Pudlak syndrome and Menkes disease with 88% and 94% efficiency, respectively (Shen *et al.*, 2018). If CROTON can predict specific DNA sequences resulting from template-free repair of a CRISPR/Cas9-induced DSB, it may also be utilized to design gRNAs capable of restoring normal gene function.

Furthermore, CROTON may be adapted to elucidate the fundamental cellular and molecular alterations induced by CRISPR/Cas9 editing. CROTON can be used to form a deep learning pipeline with existing algorithms that can predict transcription, splicing and polyadenylation from raw DNA sequences (Bogard *et al*., 2019; Jaganathan *et al*., 2019; Zhou *et al*., 2018). This CROTON-based platform would allow CRISPR/Cas9 to be used for precise manipulation of the transcriptome, thus creating a novel paradigm for functional genomics and biomedicine.

## Supplementary Material

Supplementary Data
