# Supplementary material for: CROTON: an automated and variant-aware deep learning framework for predicting CRISPR/Cas9 editing outcomes
Source: Bioinformatics. 2021 Jul 12;37(Suppl 1):i342–8. doi: 10.1093/bioinformatics/btab268 (PMC8275342; doi:10.1093/bioinformatics/btab268)
Supplement: Supplementary Data [file btab268_supplementary_data.pdf]

Supplementary Tables 1–4: The top ten gRNA targets least impacted by SNVs for *ACE2*, *CCR5*, *CTLA4*, and *PDCD1*. In the "reference sequence" column, the "NGG" PAM sites for each target location is underlined.

Table 1: The Top Ten *ACE2* gRNA Targets Least Impacted by SNVs

mean impact (%)	start	end	strand	reference sequence
0.21062151	15561932	15561992	–	AGAAAATCCTTATGCCTCCATCGATATTAGCAAAGGAGAAAATAATCCAGGATTCCAAAA
0.27175315	15581218	15581278	–	ATGTTCAGAAAGCAGTCTGCCATCCCACAGCTTGGGACCTGGGGAAGGGCGACTTCAGGT
0.30854053	15581289	15581349	–	CTTTGTATCTGTTGGTCTTCCTAATATGACTCAAGGATTCTGGGAAAATTCCATGCTAAC
0.32200278	15575706	15575766	–	GGGACTCTGCCATTTACTTACATGTTAGAGAAGTGGAGGTGGATGGTCTTTAAAGGGGAA
0.36593423	15581218	15581278	+	ACCTGAAGTCGCCCTTCCCCAGGTCCCAAGCTGTGGGATGGCAGACTGCTTTCTGAACAT
0.38006856	15566337	15566397	–	TGAAGATGTTTGTTTTGTTTCTCTACAGGGAGGAGGATGTGCGAGTGGCTAATTTGAAAC
0.38217225	15578185	15578245	+	GAATCCTTCATTAGCTCCATTTCTTAGCAGAAAAGGTTGTGCAGCATATGCCATATCATA
0.38414068	15591700	15591760	–	TATATGAAGAGTATGTGGTCTTGAAAAATGAGATGGCAAGAGCAAATCGTAAGTTTGCTG
0.38504319	15570310	15570370	+	GCTGATTTTAGGCTTATCCTCACTTTGATGCTTTGGTCTGCATCTGATTAAAGAGAAATA
0.38858168	15587816	15587876	+	TGCCCTCACATAGGCATGAAGATGTTCATATAATGGTTTAATCTGAAAAGCCCAGGAAGA

Table 2: The Top Ten *CCR5* gRNA Targets Least Impacted by SNVs

mean impact (%)	start	end	strand	reference sequence
0.36914726	46373897	46373957	+	CGAGCGAGCAAGCTCAGTTTACACCCGATCCACTGGGGAGCAGGAAATATCTGTGGGCTT
0.42917599	46373896	46373956	–	AGCCCACAGATATTTCCTGCTCCCCAGTGGATCGGGTGTAAACTGAGCTTGCTCGCTCGG
0.43764766	46373897	46373957	–	AAGCCCACAGATATTTCCTGCTCCCCAGTGGATCGGGTGTAAACTGAGCTTGCTCGCTCG
0.48346486	46373657	46373717	–	GAGCTACTGCAATTATTCAGGCCAAAGAATTCCTGGAAGGTGTTCAGGAGAAGGACAATG
0.48773587	46373672	46373732	–	TGGTCCAACCTGTTAGAGCTACTGCAATTATTCAGGCCAAAGAATTCCTGGAAGGTGTTC
0.49421897	46373898	46373958	+	GAGCGAGCAAGCTCAGTTTACACCCGATCCACTGG

				GGAGCAGGAAATATCTGTGGGCTTG
0.55001562	46373384	46373444	+	CCCAGGAATCATCTTTACCAGATCTCAAAAAGAAGGTCTTCATTACACCTGCAGCTCTCA
0.57633602	46373562	46373622	+	CTGCTTCGGTGTCGAAATGAGAAGAAGAGGCACAGGGCTGTGAGGCTTATCTTCACCATC
0.60648068	46373493	46373553	–	GATTCCCGAGTAGCAGATGACCATGACAAGCAGCGGCAGGACCAGCCCCAAGATGACTAT
0.60923191	46372997	46373057	+	CCTGCCTCCGCTCTACTCACTGGTGTTCATCTTTGGTTTTGTGGGCAACATGCTGGTCAT

Table 3: The Top Ten *CTLA4* gRNA Targets Least Impacted by SNVs

mean impact (%)	start	end	strand	reference sequence
0.3248549	203870892	203870952	–	GTGAAATGGCTTTGCTCACCAATTACATAAATCTGGGTTCCGTTGCCTATGCCCAGGTAG
0.41831277	203870857	203870917	–	GGTTCCGTTGCCTATGCCCAGGTAGTATGGCGGTGGGTACATGAGCTCCACCTTGCAGAT
0.43518746	203870768	203870828	–	CTTGGATAGTGAGGTTCACTTGATTTCCACTGGAGGTGCCCGTGCAGATGGAATCATCTA
0.46663834	203870891	203870951	–	TGAAATGGCTTTGCTCACCAATTACATAAATCTGGGTTCCGTTGCCTATGCCCAGGTAGT
0.4799203	203870612	203870672	–	CTTTGCCTGGAGATGCATACTCACACACAAAGCTGGCGATGCCTCGGCTGCTGGCCAGTA
0.53582691	203870742	203870802	–	CCACTGGAGGTGCCCGTGCAGATGGAATCATCTAGGAAGGTCAACTCATTCCCCATCATG
0.53905856	203870631	203870691	+	CATCGCCAGCTTTGTGTGTGAGTATGCATCTCCAGGCAAAGCCACTGAGGTCCGGGTGAC
0.53929947	203870871	203870931	+	GCTCATGTACCCACCGCCATACTACCTGGGCATAGGCAACGGAACCCAGATTTATGTAAT
0.54683834	203870800	203870860	+	GGAAATCAAGTGAACCTCACTATCCAAGGACTGAGGGCCATGGACACGGGACTCTACATC
0.54927571	203871379	203871439	+	CCAGAACCGTGCCCAGATTCTGACTTCCTCCTCTGGATCCTTGCAGCAGTTAGTTCGGGG

Table 4: The Top Ten *PDCD1* gRNA Targets Least Impacted by SNVs

mean impact (%)	start	end	strand	reference sequence
0.23807455	241851162	241851222	+	AGGAAAGACAATGGTGGCATACTCCGTCTGCTCAGGGACACAGGGCACGGGGGGCTCCGG
0.28203322	241852908	241852968	+	CGTTGTCCCCTTCGGTCACCACGAGCAGGGCTGGGGAGAAGGTGGGGGGGTTCCAGGGCC
0.30129467	241852633	241852693	+	CTCTGCCCGCAGGCTCTCTTTGATCTGCGCCTTGGG

				GGCCAGGGAGATGGCCCCACAGAG
0.3746561	241852851	241852911	+	GGTACCAGTTTAGCACGAAGCTCTCCGATGTGTTGGAGAAGCTGCAGGTGAAGGTGGCGT
0.40023335	241852321	241852381	–	CTAACCCCTGACCTTTGTGCCCTTCCAGAGAGAAGGGCAGAAGTGCCCACAGCCCACCCC
0.40333828	241852851	241852911	–	ACGCCACCTTCACCTGCAGCTTCTCCAACACATCGGAGAGCTTCGTGCTAAACTGGTACC
0.4309025	241852906	241852966	+	GGCGTTGTCCCCTTCGGTCACCACGAGCAGGGCTGGGGAGAAGGTGGGGGGGTTCCAGGG
0.4445281	241852638	241852698	–	CCTACCTCTGTGGGGCCATCTCCCTGGCCCCCAAGGCGCAGATCAAAGAGAGCCTGCGGG
0.46969965	241851228	241851288	–	CCCTCAGCCGTGCCTGTGTTCTCTGTGGACTATGGGGAGCTGGATTTCCAGTGGCGAGAG
0.47102948	241852696	241852756	–	CCCAACGGGCGTGACTTCCACATGAGCGTGGTCAGGGCCCGGCGCAATGACAGCGGCACC
